# Too much or too little step width variability is associated with a fall history in older persons who walk at or near normal gait speed

**DOI:** 10.1186/1743-0003-2-21

**Published:** 2005-07-26

**Authors:** Jennifer S Brach, Jaime E Berlin, Jessie M VanSwearingen, Anne B Newman, Stephanie A Studenski

**Affiliations:** 1 University of Pittsburgh, Department of Physical Therapy, 6035 Forbes Tower, Pittsburgh, PA 15260, USA; 2 University of Pittsburgh, Department of Epidemiology, 6035 Forbes Tower, Pittsburgh, PA 15260, USA; 3 University of Pittsburgh, Division of Geriatric Medicine, 6035 Forbes Tower, Pittsburgh, PA 15260, USA

## Abstract

**Background:**

Decreased gait speed and increased stride time, stride length, double support time, and stance time variability have consistently been associated with falling whereas step width variability has not been strongly related to falls. The purpose was to examine the linear and nonlinear associations between gait variability and fall history in older persons and to examine the influence of gait speed.

**Methods:**

Gait characteristics and fall history were obtained in 503 older adults (mean age = 79; 61% female) participating in the Cardiovascular Health Study who could ambulate independently. Gait characteristics were recorded from two trials on a 4 meter computerized walkway at the subject's self-selected walking speed. Gait variability was calculated as the coefficient of variation. The presence of a fall in the past 12 months was determined by interview. The nonlinear association between gait variability and fall history was examined using a simple three level classification derived from the distribution of the data and from literature based cut-points. Multivariate logistic regression was used to examine the association between step width variability (extreme or moderate) and fall history stratifying by gait speed (1.0 m/s) and controlling for age and gender.

**Results:**

Step length, stance time, and step time variability did not differ with respect to fall history (p > .33). Individuals with extreme step width variability (either low or high step width variability) were more likely to report a fall in the past year than individuals with moderate step width variability. In individuals who walked ≥ 1.0 m/s (n = 281), after controlling for age, gender, and gait speed, compared to individuals with moderate step width variability individuals with either low or high step width variability were more likely to have fallen in the past year (OR and 95% CI 4.38 [1.79–10.72]). The association between step width variability and fall history was not significant in individuals who walked < 1.0 m/s (n = 224).

**Conclusion:**

Extreme (either too little or too much) step width variability is associated with falls in the past year in older persons who walk at or near normal gait speed and not in older persons who walk slowly (<1.0 m/s).

## Background

Variability of gait can be quantified using both temporal and spatial gait characteristics. Variability of temporal characteristics such as stride time, double support time and stance time and spatial characteristics such has stride length has been consistently associated with falling, with increased variability being associated with fall risk [1-3]. The association between step width variability and fall risk has been inconsistent. Gabell and Nayak suggest that step width is related to balance control and that an increase in step width will lead to greater stability, a possible compensation for instability [4]. In bivariate analysis, step width variability was related to falls with individuals who had fallen demonstrating reduced variability in stride width; however in multivariate analyses the association between step width variability and future falls was not significant [3]. Given the conflicting findings on step width variability and the belief that step width is believed to be related to balance control [4], we feel that it is important to further investigate the association between step width variability and fall risk.

When examining the association between gait variability and falls the influence of gait speed on the relation has often not been considered. Most of the research examining the association between gait variability and falls has either been conducted in older persons walking at a near normal walking speed [1] or has not included the effects of walking speed on the association between gait characteristics and falls [3]. Even though there is some evidence to suggest that reduced gait speed is more strongly associated with fear of falling than risk of falling [3], several studies have reported that older persons who walk slowly are at risk for falling [5-9]. However, in individuals who are walking at a near normal walking speed gait variability has been shown to be a stronger indicator of fall risk than gait speed [1]. Gait variability appears to be an early indicator of fall risk in highly mobile older persons. However, whether gait variability, specifically step width variability, provides useful information about fall risk in people who walk slowly is unknown. Therefore the purpose of this manuscript is two-fold: 1) to examine the linear and nonlinear association between gait variability (especially step width variability) and fall history and 2) to examine the influence of gait speed on the association between gait variability and fall history. We hypothesized that in people who walk at a near normal gait speed that step width variability, a gait characteristic believed to be related to balance control, would be a better indicator of falls than step length variability, a gait characteristic related to the automatic stepping pattern [4]. We believe that individuals walking at a near normal walking speed are less likely to have disruption of the automatic stepping pattern and therefore are less likely to have increased step length variability (i.e. making step length variability an unlikely indicator of falls in people who walk at a near normal walking speed). Step width which is related to balance control and not so much the automatic stepping pattern, is more variable in people walking at a normal speed, thus making it a potential indicator of fall risk in people walking at a near normal walking speed [10].

## Methods

This is a cross-sectional study of the association between gait variability and fall history in community-dwelling older adults. Measures of gait characteristics and fall history were obtained during a single clinic visit.

### Subjects

A sample of ambulatory older adults was recruited from the Pittsburgh site of the Cardiovascular Health Study (CHS), a population-based, ongoing longitudinal multi-center study of coronary heart disease and stroke risk in community-dwelling older adults age 65 years and older [11,12]. At the initiation of the CHS in 1989–90, individuals were identified from the Health Care Financing Administration sampling frame. Individuals who were 65 years or older, noninstitutionalized, expected to remain in the area for 3 years and able to give informed consent were included in the study. Individuals who were wheelchair-bound in the home or were receiving hospice care, radiation therapy or chemotherapy for cancer were excluded [11,12]. In 1989–90 an original cohort of 5201 predominately Caucasian (i.e. > 95% Caucasian) men and women were enrolled, and in 1992–93 a cohort of 687 African Americans was added.

Subjects included in the analyses were men and women from the 1998–99 clinic visit of CHS at the Pittsburgh site. Subjects at the Pittsburgh site who could walk without the assistance of another person, who did not use an assistive device for ambulation, and who could follow directions to complete the gait assessment were included (n = 503).

### Measures

#### Gait Characteristics

The GaitMat II™ system was used for the gait analysis [13]. The GaitMat II™ consists of a 4-meter long walkway on which the subject walks and a computer system which controls the GaitMat II™ and analyzes the data. In addition to the 4-meter long walkway, there are initial and final one meter inactive sections to allow for acceleration and deceleration of the participant. The GaitMat II™ is an automated gait analysis system based on the opening and closing of pressure sensitive switches when the participant walks on the walkway. After two practice passes on the GaitMat, each subject completed two passes on the GaitMat II at their self-selected walking speed for data collection.

We were primarily interested in gait speed and variability of step length, step width, step time, and stance time. Step length and width represent spatial characteristics in two different planes. Step time and stance time were selected as the temporal gait characteristics since they have been widely studied by other investigators. Gait speed was determined by dividing the time between the first and last switch closure by the distance traversed. Step length was determined as the distance between two consecutive footprints, measured from the heel of one footprint to the heel of the next footprint. Step width was determined as the distance between the outer most borders of two consecutive footprints. Step time was determined as the time between initial foot-floor contact of one foot to the initial foot-floor contact of the contralateral side for two consecutive steps. Stance time was determined as the time the foot was in contact with the floor (i.e. from initial foot-floor contact until final foot-floor contact).

Gait variability was expressed as the coefficient of variation (CV) which is SD/mean × 100. The CV for each step length, step width, step time, and stance time was calculated using two passes on the GaitMat. Prior testing showed no difference in right and left step CV, so both were used to calculate the CV [10].

#### Fall History

Fall history over the past 12 months was obtained through a structured interview. Participants were asked the following: "During the past year, have you had a fall? (Do not include falls during skiing, skating, or other activities, such as walking on ice that may affect balance.)" Participants, who reported a fall, were then asked to report the number of falls in the past year.

#### Data Analysis

Prior to data analyses the gait variability data were examined for normality. The gait variability data were relatively normally distributed (mean values approximately equal to median, low values for skewness and kurtosis). For step width variability there were a few extreme outliers with individuals having high values for step width variability. The raw data were visually examined to make sure there was not an error in data collection. The values were accurate and were attributed to individuals crossing one foot over the other when walking. Since these high values represented a natural phenomenon and were not due to data collection error they were retained in the analyses. Independent t-tests were used to compare the gait variability data (step width, step length, stance time, and step time) between the individuals who had reported a fall in the past year and those who did not report a fall in the past year for the entire sample and then stratifying by walking speed (less than or greater than or equal to 1.0 m/s) [5,14-16].

After discovering the large standard deviation associated with the step width variability measure, we decided to explore if the association between step width variability and fall history was nonlinear. A simple four level classification (quartiles) was used to explore the potential nonlinear association between step width variability and fall history. Since the range of values for the lowest and highest quartile was much larger than the range of values for the middle two quartiles we decided to explore the association using a 10 level classification (deciles). Once again, the range of values for the lowest and highest deciles were much larger than the range of values for the middle 8 deciles so we further divided the sample by examining the lowest and highest 5% of the sample in regards to step width variability. The classification of step width variability was collapsed into three groups: low step width variability (step width variability CV < 7%; lowest 5% of sample), moderate step width variability (step width variability CV = 7–30%; middle 90% of sample), and high step width variability (step width variability CV > 30%; highest 5% of the sample). Fall history (% fallen in the past year) was compared across the groups using chi-square tests for the entire sample and then stratifying by gait speed (less than or greater than or equal to 1.0 m/s).

A series of logistic regression models were used to examine the association between step width variability and fall history. The first model examined the bivariate association between step width variability and fall history. The second model controlled for age and gender, and the third model accounted for gait speed. The series of models was calculated for the entire sample and then stratifying the sample by gait speed (less than or greater than or equal to 1.0 m/s). An interaction between gait speed and step width variability was also examined in the entire sample. Initially, step width variability was examined as a 3 level variable (low, moderate and high). However, after stratifying the sample by gait speed the numbers of subjects in the low and high step width variability groups were low (gait speed ≥ 1.0 m/s and low step width variability n = 3; gait speed < 1.0 m/s and high step width variability n = 9) so the models are presented with step width variability as a dichotomous variable with step width either being extreme (low or high) or moderate.

## Results

Eighty-one (16%) of the 503 participants reported experiencing one or more falls in the past year (32 reported falling more than once). Individuals who had reported a fall in the past year were slightly older, and more likely to be female than individuals who had not fallen in the past year (Table 1). The average gait speed for the sample was 1.03 m/s (SD = .21).

**Table 1:** Characteristics of entire study cohort and stratifying by past year fall history

	Total sample N = 503	No falls N = 422	≥1 fall N = 81	P-value*
Age (years)	79.3 (4.1)	79.1 (3.9)	80.3 (5.1)	.02
Weight (lbs)	154.9 (28.9)	154.8 (28.3)	155.0 (32.4)	.96
Height (cm)	164.3 (9.3)	164.5 (9.4)	163.0 (9.0)	.18
ADL difficulty n (%)	148 (29.4)	122 (28.9)	26 (32.1)	.56
African American n (%)	113 (22.5)	93 (22.0)	20 (24.7)	.80
Female n (%)	306 (60.8)	248 (58.8)	58 (71.6)	.03
Gait Characteristics				
Step width (m)	0.21 (.04)	0.22 (0.04)	0.21 (0.05)	.46
Step length (m)	0.57 (0.09)	0.57 (0.09)	0.56 (0.10)	.14
Stance time (s)	0.73 (0.09)	0.73 (0.09)	0.73 (0.10)	.71
Step time (s)	0.56 (0.06)	0.56 (0.06)	0.56 (0.06)	.81
Gait speed (m/s)	1.03 (0.21)	1.04 (.20)	1.02 (.23)	.40

* P-values are for independent t-test comparing continuous data and chi square comparing categorical data between individuals who reported no falls and those who reported ≥ 1 fall in the past year.

In the entire sample, individuals who had reported a fall in the past year did not differ on any of the measures of gait variability when compared to individuals who had not reported a fall (Table 2). Likewise, gait speed did not differ between individuals who reported and did not report a fall in the past year. When stratifying the sample by gait speed, individuals who walked faster than 1.0 m/s and reported a fall in the past year were more variable in step width than individuals who had not fallen in the past year (Table 3). In individuals who walked slowly (< 1.0 m/s) gait variability did not differ with respect to fall history.

**Table 2:** Mean (SD) Gait Characteristics stratifying by past year fall history

	No falls N = 422	≥ 1 fall N = 81	p-value
Gait Variability CV* (%)			
Step width	17.8 (16.4)	21.8 (22.6)	.06
Step length	6.3 (3.0)	6.5 (3.1)	.62
Stance time	4.9 (2.0)	5.2 (2.1)	.33
Step time	4.7 (1.8)	4.7 (1.8)	.90

*CV = coefficient of variation

**Table 3:** Mean (SD) gait characteristics stratifying by gait speed and past year fall history.

	Gait Speed < 1.0			Gait Speed ≥ 1.0 m/s		
	No falls n = 185	≥ 1 fall n = 37	p-value	No falls n = 237	≥ 1 fall n = 44	p-value
Gait Variability CV* (%)						
Step width	15.6 (15.9)	15.7 (7.7)	.95	19.6 (16.6)	26.8 (29.1)	.02
Step length	7.5 (3.4)	7.7 (3.7)	.77	5.4 (2.3)	5.5 (2.2)	.77
Stance time	5.7 (2.3)	6.0 (2.3)	.47	4.3 (1.5)	4.5 (1.5)	.55
Step time	5.4 (1.7)	5.3 (2.1)	.83	4.1 (1.6)	4.2 (1.3)	.79
						
Gait speed (m/s)	.85 (.11)	.82 (.14)	.11	1.18 (.13)	1.18 (.14)	.98

*CV = coefficient of variation

**Figure 1:**
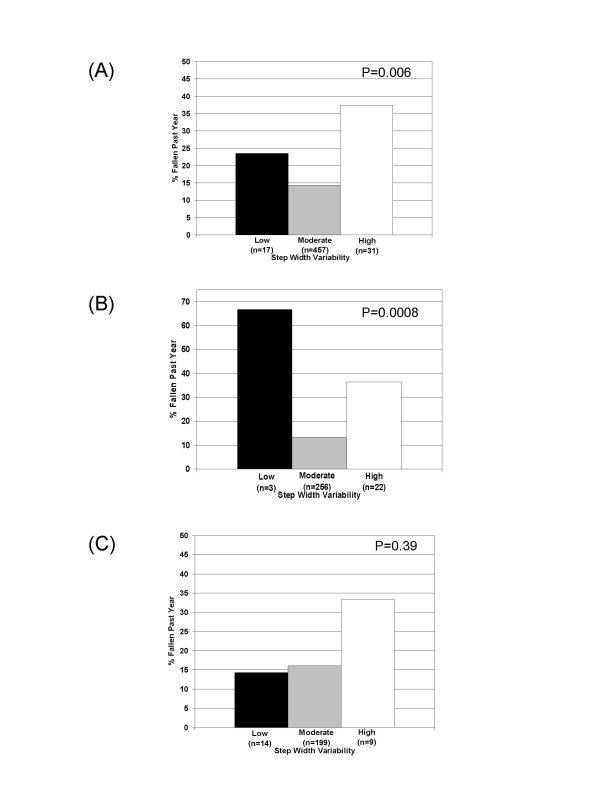
Percent of participants who reported a fall in the past year by amount of step width variability (low, moderate, or high) for (A) the entire sample, (B) individuals with a gait speed ≥ 1.0 m/s, and (C) individuals with a gait speed < 1.0 m/s (C). P-values are for Chi-square.

There was no association between step length, step time and stance time variability groupings (quartiles or cut-point based groupings) and fall history (p > .25). Step width variability examined as a three level categorical variable (low, <7%; moderate 7–30%; or high, > 30%) was associated with fall history in the entire sample (Figure 1A). Individuals with extreme step width variability (either low or high) were more likely to report a fall in the past year than individuals with moderate step width variability (p = .006); thus providing evidence that the association between fall history and step width variability may be nonlinear. The association remained in individuals who walked faster than 1.0 m/s, p = .0008 (Figure 1B), but was not significant in those who walked slowly, p = .39 (Figure 1C). The lack of significant findings in those individuals who walked slowly could possibly be attributed to inadequate power due to the small sample size in the extreme step width variability groups (low step width variability n = 14 and high step width variability n = 9).

In the entire sample, after controlling for age, gender, and gait speed, compared to individuals with moderate step width variability individuals with low or high step width variability were 2.76 times more likely to have fallen in the past year (OR and 95% CI were 2.76 [1.40–5.45]). In individuals who walked ≥ 1.0 m/s (n = 281), after controlling for age, gender, and gait speed, compared to individuals with moderate step width variability individuals with either low or high step width variability were 4.38 times more likely to have fallen in the past year (OR and 95% CI were 4.38 [1.79–10.72]). The association between step width variability and fall history was not significant in individuals who walked < 1.0 m/s (Table 4). In the entire sample, the interaction term between gait speed and step width variability was not significant (p = .16).

**Table 4:** Association of step width variability with past year fall history: Odds Ratios (95% Confidence interval) and Adjusted Odds Ratios (95% Confidence interval) for age, gender and gait speed.

	Model 1	Model 2	Model 3
**Total Sample n = 503**			
Mod step width CV* N = 455	1	1	1
Low/High Step width CV N = 48	2.65 (1.36, 5.14)	2.70 (1.37, 5.31)	2.76 (1.40, 5.45)
Age (years)		1.08 (1.02, 1.14)	1.08 (1.02, 1.15)
Gender		.55 (.33, .93)	.51 (.30, .89)
Gait speed (m/s)			1.32 (.38, 4.63)

**Speed < 1.0 m/s N = 222**			
Mod Step width CV N = 199	1	1	1
Low/High Step width CV N = 23	1.42 (.49, 4.08)	1.56 (.52, 4.70)	1.56 (.50, 4.79)
Age (years)		1.08 (1.00, 1.17)	1.08 (.99, 1.17)
Gender		.42 (.17, 1.02)	.38 (.15, .99)
Gait speed (m/s)			.22 (.01, 4.33)

**Speed ≥ 1.0 m/s N = 281**			
Mod Step width CV N = 256	1	1	1
Low/High Step width CV N = 25	4.35 (1.81, 10.47)	4.38 (1.79, 10.72)	4.38 (1.79, 10.72)
Age (years)		1.09 (1.00, 1.18)	1.09 (1.00, 1.19)
Gender		.67 (.33, 1.33)	.65 (.32, 1.31)
Gait speed (m/s)			1.62 (.12, 21.11)

*CV = coefficient of variation

## Discussion

This study of gait variability and fall history in community-dwelling older individuals has two key findings. First, among community-dwelling older persons ambulating independently the association between step width variability and fall history was nonlinear. Consistently it has been shown that increased variability of stride length, stride time, and stride speed are related to falls [2,3,17], where as decreased step width variability has been only associated with falls during walking in one research report [3]. The lack of significant findings associating step width variability with fall history may be the result of assuming a linear relationship between step width variability and falls. For step width variability the normal situation is to have a moderate amount of variability. We discovered that not only having too little step width variability but also having too much step width variability was associated with a history of falls.

Gabell and Nayak suggest that step width is related to balance control and that an increase in step width variability could indicate a lack of compensation for instability [4]. Both young and older person who have not fallen demonstrate an increased level of step width variability (median step width variability in young and old is 20.62 and 26.84, respectively) compared to variability of other gait characteristics such as step length (median step length variability in young and old is 5.41 and 5.31, respectively) and stride time (median stride time variability in young and old is 2.60 and 3.21, respectively), suggesting that a moderate amount of step width variability is required to adapt to the situation and to "stay on ones feet" [3,4]. Individuals who are unable to vary their step width (i.e. individuals with low step width variability) may be lacking the skills necessary to adjust their step width to maintain their balance. On closer inspection of the raw gait data of individuals who vary their step width a lot (i.e. individuals with high step width variability), high step width variability was often associated with crossing one foot over the other during walking, a gait deviation that clinically is indicative of unsteady walking.

The second major finding is that step width variability was associated to fall history only in people walking at a near normal walking speed (>1.0 m/s) [5,14-16]. Gait speed has previously been associated with fall status [5,6,9,18]. In people who walk slowly, fall risk may be due to numerous abnormalities independent of step width variability[7,19-23]. Our findings suggest that in individuals who are not identified to be at risk for falls by their gait speed (i.e. those walking at a near normal gait speed) variability of step width may provide valuable information about fall risk. In people walking at a near normal walking speed, some degree of step width variability may be adaptive where too little or excessive step width variability on a simple non-challenging mat surface may be abnormal. Excessive step width variability in a non-challenging situation (i.e. where adaptation is not necessary) could potentially be an early indicator of fall risk in highly mobile people.

Our findings are somewhat conflicting to other research examining gait variability and falls. Specifically, we found no association between fall history and variability of step length, stance time and step time where others have shown an association with similar gait characteristics[1-3,17]. One potential explanation may be the way the gait characteristics were measured in this study. Gait variability was calculated from a limited number of steps, in most cases less than twelve steps. Others that have shown an association between stride time and swing time variability have calculated variability using data from hundreds if not thousands of steps [1,2,24,25]. Since the two methodologies, gait mat and pressure sensitive insoles, have yet to be directly compared, we do not know if similar information regarding gait variability is obtained. A major strength of the methodology used was the ability to examine step width, a spatial gait characteristic. The methodologies that capture hundreds/thousands of steps are based on temporal measures of gait and do not record the spatial characteristics such as step width. Our methodology was similar to that used in the research by Maki where they showed an association between stride length, double support time, and stride velocity variability and future falls [3]. The discrepancy in findings may be partly explained by the fact that on average both our total sample and our sub-sample of individuals walking slowly (i.e. < 1.0 m/s) were walking faster then their sample (mean gait speed our total sample = 1.03 m/s, mean gait speed our sub-sample = 0.85 m/s, mean gait speed Maki's sample = 0.70 m/s).

Finally, it is important to note that we examined the association of gait variability to falls over the past year where others have look at the association with future falls [2,3]. In actuality, the participants' gait was measured after the person had fallen. Having experienced a fall, the participant may have changed the way they walked, possibly walking slower since fear of falling is related to gait speed [3], thus influencing the results. Classification of fall status was based on the participants' remembering if they had fallen during the past year. One of the limitations of using past year fall status is the likelihood of recall error which may lead to misclassification of the sample. Ideally, we would have preferred to examine the association between recurrent falls (i.e. falling 2 or more times in the past year) and gait variability; however, only a limited number of participants experienced more than one fall (n = 32). When examining gait variability between individuals who had not fallen in the past year and those who had reported falling two or more times in the past year the results were similar to those reported; however they are not presented given the limited power of the analyses.

It is important to note that the older persons included in this study are relatively healthy. For example, the fall rate of the sample (16%) is much lower than what is traditionally reported for community-dwelling older persons. Also, in order to be included in this study the individual had to be a participant of an ongoing research study (i.e. CHS), travel to the research clinic of the study examination, and had to be able to walk independently without an assistive device, all criteria that could potentially bias the sample towards being healthier than the average community-dwelling older person. However, this sample is ideal for examining early indicators for fall risk that occur prior to assistive device use and decreases in gait speed.

## Conclusion

Extreme step width variability (i.e. either too much or too little) is associated with fall history in older adults walking at or near normal walking speed. Further research is required to determine if extreme step width variability is a predictor of falls in older persons without mobility limitations.

## Competing interests

The author(s) declare that they have no competing interests.

## Authors' contributions

JSB participated in the design of the study, carried out the gait data collection, analyzed the data, and drafted the manuscript. JEB participated in the data analyses and manuscript preparation. JVS participated in the design of the study, data analyses, and writing the manuscript. ABN conceived of the study, participated in the design of the study, data analyses and writing the manuscript. SAS participated in the data analyses and writing of the manuscript.
